# Chronic and Heavy Drinking, Nutrition Status, and Progression of Liver Injury Negatively Affect the Mortality Risk in Patients Suffering from Alcohol-Associated Hepatitis

**DOI:** 10.3390/jcm14176157

**Published:** 2025-08-31

**Authors:** Aishwarya Thakurdesai, Anjali Kumari, Henry Shay, Khaled Elgharabawy, Evan J. Winrich, Wanyu Zhang, Amber Jackson, Matthew C. Cave, Maiying Kong, Xiang Zhang, Ashwani K. Singal, Craig J. McClain, Vatsalya Vatsalya

**Affiliations:** 1 Division of Gastroenterology, Hepatology and Nutrition, Department of Medicine, University of Louisville, Louisville, KY 40202, USA; aishwarya.thakurdesai@louisville.edu (A.T.); anjali.kumari.1@louisville.edu (A.K.); henry.shay@louisville.edu (H.S.); khaled.elgharabawy@louisville.edu (K.E.); wanyu.zhang@louisville.edu (W.Z.); amber.jackson@louisville.edu (A.J.); matt.cave@louisville.edu (M.C.C.); ashwani.singal@louisville.edu (A.K.S.); craig.mcclain@louisville.edu (C.J.M.); 2 Clinical Laboratory for the Intervention Development of AUD and Organ Severity, Louisville, KY 40202, USA; 3 Robley Rex VA Medical Center, Louisville, KY 40206, USA; 4 Alcohol Research Center, University of Louisville, Louisville, KY 40202, USA; maiying.kong@louisville.edu (M.K.); xiang.zhang@louisville.edu (X.Z.); 5 Department of Bioinformatics and Biostatistics, University of Louisville, Louisville, KY 40202, USA; 6 Center for Regulatory and Environmental Analytical Metabolomics, University of Louisville, Louisville, KY 40292, USA

**Keywords:** ABIC, AUD, AH, ALD, CONUT, mortality, nutrition

## Abstract

**Background/Objectives**: Alcohol-associated hepatitis (AH) is an acute inflammatory condition of alcohol-associated liver disease (ALD) with rapid progression and high mortality. The Age-Bilirubin-INR-Creatinine (ABIC) score is a static algorithm that predicts survivability in AH. The roles of alcohol drinking patterns and nutritional status in AH progression and risk of death are understudied. This study evaluates the impact of alcohol drinking patterns and nutrition on AH progression and mortality. **Methods**: Sixty-one adult patients diagnosed with AH were stratified by the Model for End-Stage Liver Disease (MELD) as non-severe (MELD < 20, *n* = 26, Gr.1) and severe (MELD ≥ 20, *n* = 35, Gr.2). Each group was further subdivided by ABIC: low- (<6.71), intermediate- (6.71–9), and high- (>9) risk categories. We assessed different demographics: nutrition using the Controlling Nutritional Status (CONUT) score; lifetime drinking history (LTDH); recent alcohol use (AUDIT); laboratory measures (complete metabolic panel, complete blood count, and coagulation), and clinical measures (Maddrey DF, Child–Turcotte–Pugh, and Lille). **Results**: All patients showed a significant and positive correlation between ABIC and LTDH (r = 0.538, *p* = 0.004), particularly in Gr.2 (r = 0.554, *p* = 0.011). The low-risk Gr.2 exhibited the highest AST:ALTs. AST:ALTs were significantly associated with LTDH, AUDIT, and CONUT (R^2^ = 0.539, *p* = 0.031). In all AH patients with intermediate mortality risk, AST:ALTs were strongly linked to CONUT and LTDH (R^2^ = 0.657, *p* = 0.017). **Conclusions**: Severe AH demonstrates rapid liver injury progression even when the mortality risk is low. Chronic and recent heavy alcohol consumption and poor nutrition adversely impact AH severity and mortality risk. Alcohol intake and nutritional assessments in routine clinicals could identify high-risk patients, thereby improving treatment and a favorable prognosis.

## 1. Introduction

Heavy alcohol consumption can lead to liver damage, ranging from early-stage alcohol-associated liver disease (ALD) to advanced forms of ALD, including acute form, alcohol-associated hepatitis (AH), and chronic form as alcohol-associated cirrhosis [1,2]. Alcohol-associated liver disease (ALD) is the leading cause of alcohol-related deaths worldwide [3,4]. Sustained, excessive alcohol use can cause acute hepatic inflammation known as AH, one of the most severe manifestations of ALD associated with significant morbidity and mortality [3,5]. This may present with a concerning clinical profile characterized by an abrupt onset of jaundice, malaise, decompensated liver disease, and coagulopathy [6]. A subset of patients with AH will eventually develop severe AH, which is associated with bacterial infections and the development of acute-on-chronic liver failure, as well as multiorgan failure [3]. Severe AH has staggering mortality rates, ranging from 20% to as high as 50% in 3 months [7,8].

AH patients frequently exhibit extensive chronic as well as active alcohol consumption [9]. Although chronic drinking, once established, may not be modified, continued recent heavy alcohol consumption and nutrition are prospectively important modifiable clinico-pathological factors of ALD [10]. If addressed promptly and with precise medical management, this could improve the clinical course of AH, leading to improved outcomes and survival [11]. Furthermore, these patients are often malnourished, with almost all patients with severe AH demonstrating some degree of malnutrition [12,13]. Although substantial alcohol consumption among these patients becomes a major source of calories, these should be considered “empty” calories that contain little nutritional value [14,15]. AH patients frequently experience insufficient protein intake and may have an imbalanced fat intake, characterized by an excess of omega-6 and a deficiency of omega-3 [16,17]. Additionally, their diets often lack essential micronutrients, such as zinc and magnesium [4,18,19]. Consequently, alcohol consumption and poor nutrition intersect differentially, contributing to the development of AH and its associated complications. Malnutrition in AH is associated with complications such as hepatic encephalopathy and worse liver outcomes [20]

The primary therapy for AH is the cessation of alcohol use and supportive care [21,22]. Despite extensive studies, specific medical therapies are limited and still rely on the use of glucocorticoids, which improve short-term (30-day) but not long-term survival in selected patients [3,6,21,23]. Aggressive nutritional support remains the cornerstone of supportive care in AH [21]. Its role could be useful in the assessment of prognostic outcomes. The role of chronic and recent drinking and nutritional status in the progression of liver injury and mortality in AH remains a research gap.

A few scoring models have been developed to determine the severity of liver disease, predict mortality, and guide treatment protocols in AH. The Model for End-Stage Liver Disease (MELD) score is among the oldest scoring systems for AH. The original MELD score consists of serum bilirubin and creatinine levels, the International Normalized Ratio (INR) for prothrombin time, and the etiology of liver disease [24]. MELD scores > 20 have been used to identify severe disease and as a threshold to start corticosteroid therapy in AH [3,24,25]. The ABIC score is a newer algorithm that allows the stratification of the risk of death in patients with AH. This study aims to evaluate chronic and severity assessments of alcohol drinking and nutritional status in AH patients at severe and non-severe stages. We also aimed at investigating the interplay of these measures and their contributing role in the mortality risk (ABIC) in AH patients.

## 2. Materials and Methods

The study participants were enrolled under the clinical study, which was approved by the IRB of the University of Louisville under protocol # 94.0261 (UofL Biorepository). All eligible patients signed the study consent form to participate in this investigation before any data were collected. This clinical investigation was performed on 61 individuals aged 21–68 years. Out of the 61 individuals, 26 individuals with non-severe alcohol-associated hepatitis were diagnosed according to the clinical and laboratory guidelines, as developed recently by the National Institute on Alcohol Abuse and Alcoholism (NIAAA) consortium [26]. The Model for End-Stage Liver Disease (MELD) [27] was used as the differential criterion to allocate the participants as non-severe (MELD < 20, *n* = 26) and severe (MELD ≥ 20, *n* = 35). Additionally, the data from this clinical investigation were also analyzed with the perspective of ABIC as a primary factor of discrimination of risk (ABIC < 6.71, *n* = 16; 9 ≤ ABIC > 6.71, *n* = 36; and ABIC ≥ 9, *n* = 9).

### 2.1. Clinical Measures, Markers, and Determinants

The demographic factors included age, which was collected over the years. Age was in increasing order on the risk assessment as determined by ABIC. BMI was in the obese category in both the severe and non-severe patients [28]. Sex was categorized as male and female by the individuals’ identification with their birth sex. Race was largely limited to Caucasians, primarily, and African American enrollees.

Drinking assessments were performed using the AUDIT and lifetime drinking history clinical batteries. Alcohol consumption history for the past year was assessed using the Alcohol Use Disorders Identification Test (AUDIT) [29] for the previous year and the lifetime drinking history (LTDH) for cumulative exposure from the beginning of drinking (in years with epoch illustrations) [30]. Nutritional status was evaluated using a screening tool called the Controlling Nutritional Status (CONUT) score [31]. The score assesses the nutritional status of those who undergo routine clinical laboratory analysis (using categorical values from the albumin from the comprehensive metabolic panel (CMP), absolute lymphocytes from the complete blood count (CBC), and total cholesterol (in lipid panel) readings).

The primary liver clinical markers assessed were alanine transaminase (ALT), aspartate transaminase (AST), total bilirubin (TBili), and albumin (ALB) using the clinical laboratory testing for the liver panel, as well as for the International Normalized Ratio value (INR) using coagulation testing. The following descriptions provide liver severity ratings. An algorithm to predict mortality in alcohol-associated hepatitis (AH) was used as a primary study theme, using age, total bilirubin, INR, and creatinine (ABIC score as a unit as well as a categorical score). The risk of death in AH is further classified into low (ABIC score < 6.71), intermediate (ABIC score 6.71–8.99), and high (ABIC score > 9.0) using the ABIC score. A 90-day mortality risk of 0%, 30%, and 75%, respectively, is associated with these classes (1, 10). The other liver-associated severity indices that were used are as follows: MELD—Model for End-Stage Liver Disease [27], Maddrey-DF—Maddrey’s Discriminant Function [32], CTP—Child–Turcotte–Pugh score [33,34], Lille—Lille model for alcohol-associated hepatitis (mortality assessment at day 7) [35], and AST:ALT—Aspartate transaminase/Alanine transaminase ratio [36].

### 2.2. Analyses, Design, and Statistics

**Figure 1:**
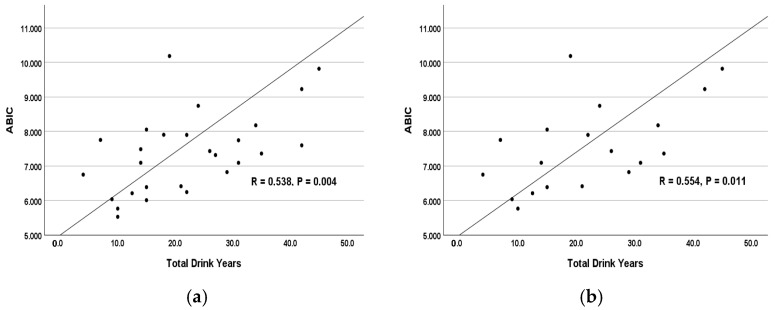
Association of ABIC score and lifetime drinking history (total drink years) in AH patients. (**a**). Relationship between ABIC and lifetime drinking history among all AH patients. (**b**). Relationship between ABIC and lifetime drinking history in severe AH patients. ABIC = Age-Bilirubin-INR-Creatinine. AH = Alcohol-associated hepatitis. The association is presented as Pearson’s correlation coefficient, R. Statistical significance is set at *p* < 0.05.

**Figure 2:**
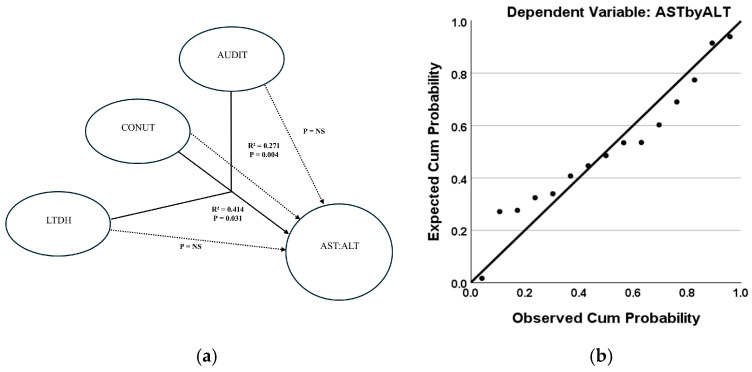
Interaction of chronic and recent heavy drinking, nutrition, and progression of liver injury in severe AH patients. (**a**). Multivariable regression model depicting the role of nutritional status and drinking characteristics in liver injury (measured by AST:ALT). The combination of LTDH, CONUT score, and AUDIT score predicts liver injury progression in severe AH patients (solid black arrow). Dotted arrows show the individual effects of each factor using univariate regression models. (**b**). A probability–probability plot demonstrates the predictability of liver injury progression (AST:ALT) by LTDH, CONUT, and AUDIT in a multivariate regression model for severe AH patients. ABIC = Age-Bilirubin-INR-Creatinine Score. AST = Aspartate transaminase. ALT = Alanine transaminase. AUDIT = Alcohol Use Disorders Identification Test score. CONUT = Controlling Nutritional Status score. LTDH = Lifetime Drinking History in years. Association is presented as the coefficient of determination, R^2^. Statistical significance is set at *p* < 0.05.

**Figure 3:**
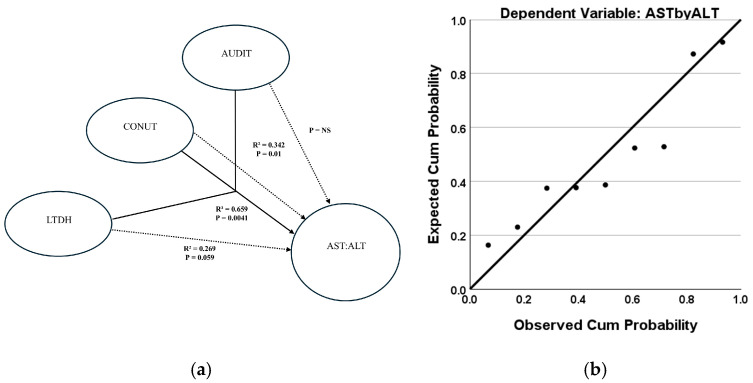
Interaction of chronic and recent heavy drinking, nutrition, and progression of liver injury in severe AH patients with intermediate mortality risk as stratified by the ABIC score. (**a**). Multivariable regression model depicting the role of nutritional status and drinking characteristics in liver injury (measured by AST:ALT). The combined effect of LTDH, CONUT, and AUDIT score predicts liver injury progression more strongly (solid black arrow) compared with severe AH patients overall. Dotted arrows show individual effects. (**b**). Probability–probability plot demonstrating the predictability of liver injury progression (AST:ALT) by LTDH, CONUT, and AUDIT in a multivariate regression model in severe AH patients with intermediate mortality risk. ABIC = Age-Bilirubin-INR-Creatinine Score. AST = Aspartate transaminase. ALT = Alanine transaminase. AUDIT = Alcohol Use Disorders Identification Test score. CONUT = Controlling Nutritional Status score. LTDH = Lifetime Drinking History in years. Association is presented as the coefficient of determination, R^2^. Statistical significance is set at *p* < 0.05.

**Figure 4:**
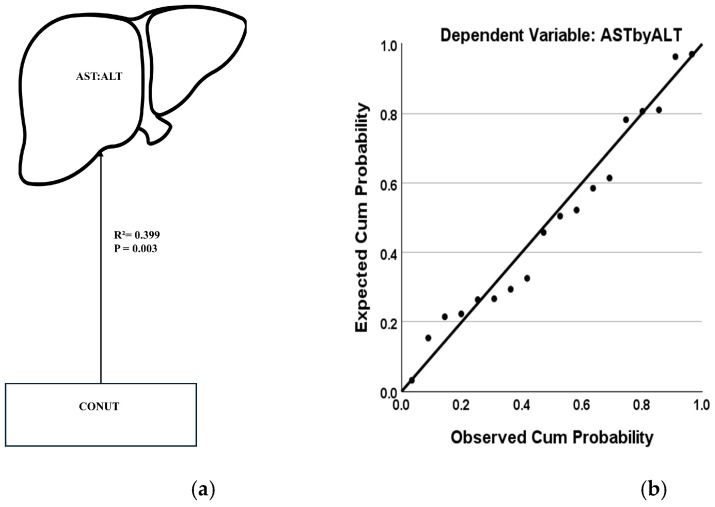
Predictability of liver injury progression (AST:ALT) by nutritional status (CONUT score) in AH patients with moderate–severe AUD or alcohol dependence (AUDIT score > 15). (**a**): Univariate regression model demonstrating the effect of nutritional status on liver injury progression. (**b**): Probability–probability plot demonstrating the predictability of liver injury progression by nutritional status. AST = Aspartate transaminase. ALT = Alanine transaminase. AUD = Alcohol Use Disorder. AUDIT = Alcohol Use Disorders Identification Test score. CONUT = Controlling Nutritional Status score. Association is presented as the coefficient of determination, R^2^. Statistical significance is set at *p* < 0.05.

This study was designed to identify the differences in the demographic, drinking, and clinical presentation of AH patients who suffer from non-severe and severe forms of advanced and acute ALD. This study also describes the clinical severity and mortality differences between the two study groups. A between-group independent samples *t*-test was used for comparing the differences, as shown in Table 1. ANOVA was used to test for overall group differences. Univariate and multivariate linear regression models (Figure 1, Figure 2, Figure 3 and Figure 4) were used to test the association and impact of the arrangement of the data, respectively. Data assembly, processing, curation, and coding were conducted using Microsoft Excel (Microsoft 365, Microsoft Corporation, Redmond, WA, USA). Data analyses were performed using the Statistical Package for Social Sciences (SPSS) version 29.0 (International Business Machines (IBM), Armonk, New York, NY, USA) and GraphPad Prism version 10.2.2 (Dotmatics, GraphPad Software, Boston, Massachusetts, MA, USA). Data analyses have been presented with model fit outcomes, using an adjusted R^2^ descriptively (mild < 0.2), (0.2 < moderate < 0.7, or high > 0.7) and corresponding F-test and RMSE (root mean square error). Statistical significance was set at a probability value (*p*-value) of <0.05. Data were presented as mean ± standard deviation (SD) unless specified otherwise.

## 3. Results

### 3.1. Demographics and Drinking Patterns

Group 1 (nSAH, MELD < 20) had 26 patients (42.62%; 18 females, 8 males), while Group 2 (SAH, MELD > 20) had 35 patients (57.38%; 13 females, 22 males). In Group 1, the low- and moderate-mortality-risk subgroups comprised 10 and 16 patients, respectively. No Group 1 patients were in the high-mortality-risk subgroup. Group 2 had 6, 20, and 9 patients in the low-, intermediate-, and high-mortality-risk subgroups, respectively.

The mean age of the participants was 48.66 ± 10.31 years. Group 2 patients were significantly older than Group 1 patients (*p* = 0.023). Age differences were significant across all five subgroups. Higher ABIC scores (greater risk of death) corresponded with increasing age in all subgroups. The average BMI of the participants was 31.83 ± 9.16. The differences in BMI were not statistically significant between groups and subgroups. A total of 8 patients were of African American descent, whereas 53 patients were of Caucasian descent.

The mean AUDIT scores of both groups were more than 15, indicating a moderate-to-severe level of alcohol dependence. AUDIT scores were not significantly different between groups or subgroups. Lifetime drinking histories (LTDH) were similar in Group 1 and Group 2. Group 1 patients with a moderate mortality risk had a numerically higher LTDH than Group 1 patients with a low mortality risk. Similarly, in Group 2, LTDH was progressively higher from the low- to intermediate- to high-mortality-risk subgroups. This difference in LTDH between the low- and high-mortality-risk subgroups of Group 2 was statistically significant (*p* = 0.017).

Mean CONUT scores were significantly higher in Group 2 patients than in Group 1 patients. Subgroup analysis further revealed additional differences between the subgroups. Remarkably, within Group 2, greater CONUT scores, corresponding to poorer nutritional status, were observed as more concerning in the low-mortality-risk subgroup compared with the high-mortality-risk subgroup (*p* = 0.046), which could be reflective of the highest AUDIT scores (severe recent drinking, resulting in empty calories) observed in this subgroup of patients. Significant differences were observed between the low-mortality-risk subgroups (*p* = 0.013) and the intermediate-mortality-risk subgroups (*p* = 0.05) of Groups 1 and 2.

Linear regression model analyses describe the role of chronic drinking in risk assessments of AH. Increasing values of the ABIC score showed a positive correlation with the corresponding increase in the length of drinking (in years), r = 0.538 at *p* = 0.004 with moderate effects (adjusted R^2^ = 0.261, F-test = 10.159, RMSE = 25.776) (Figure 1a). This correlation was stronger among the severe AH Group 2, r = 0.554 at *p* = 0.011 with moderate effects (adjusted R^2^ = 0.272, F-test = 7.714, RMSE = 19.744) (Figure 1b). Both LTDH and AUDIT are higher in this group, and the severity of AH was also around two-fold compared with the non-severe group. To note, however, this relationship was not significant in non-severe AH patients. On the other hand, recent and heavy drinking, as assessed by AUDIT alone, did not show any predictability for ABIC, either overall or within each group of AH patients. Since the severity of the AH condition has been defined by severity based on MELD (Table 1), we continued our analyses using MELD as a primary factor as above and in further sections.

### 3.2. Clinical Presentation in Severe and Non-Severe AH

Mean ALT values were mildly elevated in both groups but did not exhibit significant differences between the groups or subgroups. Mean AST levels, on the other hand, were significantly elevated in all groups and subgroups. The AST/ALT ratio was higher in Group 2 compared with Group 1, reaching a trend level of significance (*p* = 0.067). In Group 2, the highest mean AST/ALT ratio (4.17 ± 2.35) was exhibited by the lowest mortality risk subgroup. AST/ALT ratio was significantly higher in the intermediate-mortality-risk subgroup of Group 2 compared with the corresponding subgroup of Group 1 (*p* = 0.038).

Statistically significant differences were observed between the groups and subgroups regarding bilirubin and INR levels; however, these were expected as the participants were grouped based on MELD scores, which employ these variables during calculation. No differences in serum creatinine levels were observed. Group 2 exhibited significantly lower albumin levels than Group 1 (*p* < 0.001). These differences were also reflected between the low-mortality-risk subgroups (*p* = 0.011) and the high-mortality-risk subgroups (*p* = 0.007) of both groups.

Group 2 patients showed significantly higher Maddrey and CTP scores than Group 1 patients (*p* < 0.001 for both). Maddrey’s score was higher in the high-mortality-risk subgroup compared with the intermediate-mortality-risk subgroup of Group 2 (*p* = 0.017). Similar differences between subgroups of Groups 1 and 2 were observed concerning CTP scores (*p* = 0.027 for low-mortality-risk subgroups of Groups 1 and 2, *p* < 0.001 for intermediate-mortality-risk subgroups of Groups 1 and 2). Lille’s score was significantly elevated in Group 2 compared with the values reported in Group 1 (*p* = 0.043). Within Group 2, a greater Lille score in the high-mortality-risk subgroup compared with the low-mortality-risk subgroup was observed (*p* = 0.007).

Markers of hepatic synthetic function, specifically INR and albumin, also varied significantly between subgroups categorized by overall ABIC risk categories. As expected, INR was significantly elevated in the moderate -and high-risk groups, reflecting worsening liver disease. Albumin levels were significantly lower in overall high-risk males compared with their overall low-risk counterparts. Creatinine was numerically (and statistically) elevated among all three categories (low, moderate, and high overall ABIC risk) regardless of the MELD scores as well. ALT was significantly higher in males compared with their female counterparts, who had low ABIC.

### 3.3. Factors for Liver Injury Progression in AH

The multivariable regression model analyses describe the role of chronic and heavy drinking and nutritional status in risk assessments of severe AH. In the severe AH cases only, the progression of liver injury (AST:ALT) was significantly associated with chronic drinking and the observed nutritional deficiency status (CONUT score) at a moderately high effect (adjusted R^2^ = 0.405, *p* = 0.018, F-test = 5.771, and RMSE = 20.181). In this multivariable algorithm, this effect was augmented (adjusted R^2^ = 0.414, *p* = 0.031, F-test = 4.292, RMSE = 18.240) with the addition of AUDIT as another independent variable (Figure 2a). When the intermediate mortality risk subgroup of severe AH patients was similarly analyzed, the progression of liver injury (AST: ALT) could be ascertained at a highly significant effect (adjusted R^2^ = 0.657 at *p* = 0.017, F-test = 8.671, RMSE = 4.659, moderate effects) by CONUT and chronic drinking (Figure 3a).

The multivariable regression model analyses describe the role of chronic and heavy drinking and nutritional status in describing risk assessments of severe AH with an intermediate level of mortality risk. The effect size was strengthened even further with the addition of AUDIT (adjusted R^2^ = 0.654 at *p* = 0.0041, F = 6.030, RMSE = 3.925, higher end of moderate effects) (Figure 3a). This predictability of liver injury progression according to chronic drinking, nutritional deficiency, and alcohol-associated measures could be shown by probability–probability plots (Figure 2b and Figure 3b).

In non-severe AH, this analysis did not yield any conclusive significance. Remarkably, among patients exhibiting alcohol dependence (AUDIT score > 15), nutritional status as represented by CONUT had a considerable effect on liver injury progression (AST:ALT) (adjusted R^2^ = 0.427 at *p* = 0.003, moderate effects with F-test = 12.945, RMSE = 25.851) (Figure 4a). A probability–probability plot also showed this predictability (Figure 4b). A similar effect was not observed among patients without alcohol dependence.

### 3.4. Clinical Presentation of Mortality Risk in Severe AH

In severe AH patients in whom steroid treatment was indicated (Maddrey score > 32), a linear relationship of ABIC scores could be determined with AST:ALT and chronic drinking (R^2^ = 0.226, *p* = 0.050). In the low- and high-risk-mortality subgroups of severe AH, this analysis did not yield any significant results.

## 4. Discussion

This study provides valuable insights into the demographics, drinking patterns, clinical presentation, and predictive factors associated with liver injury and severity in patients with an increasing order of severity and risk assessment of mortality for alcohol-associated hepatitis (AH). This study underscores how chronic (lifetime drinking history) and heavy drinking (AUDIT—severity assessed past one year), as well as the nutritional status of the AH patients, could play a modifying role both independently and together in the course of liver injury progression and mortality risk. This investigation highlights significant associations between drinking history, declining nutritional status, and increasing liver injury severity that are highly relatable, particularly in severe AH patients. Further discussion synthesizes our findings and describes the implications derived from the data analysis.

The demographic profile of our study cohort, predominantly male in both non-severe (Group 1) and severe (Group 2) AH patients, aligns with the literature, showing a higher prevalence of AH in men [37]. Group 2 patients were older, which is consistent with the poorer prognosis associated with advanced age in liver disease [38]. While Body Mass Index (BMI) did not differ significantly between groups, chronic and heavy alcohol consumption, as reflected by high AUDIT scores, was evident across both groups, suggesting moderate to severe alcohol use disorder [39].

Our findings reveal that chronic drinking and the ABIC scores closely correlate positively with increasing order, particularly in the severe AH cases. This aligns with previous research indicating that prolonged alcohol consumption accelerates liver damage and worsens AH prognosis [40]. The sizable effect of chronic drinking on the ABIC score suggests that a longitudinal history of drinking significantly and adversely influences the progression of liver injury and, consequently, the mortality risk. This is consistent with a previous publication [41], which reported that both the duration and quantity of alcohol intake contribute to the severity of AH and its outcomes.

Our study also emphasizes the importance of nutritional status, as assessed by the CONUT score, in liver injury progression. Importantly, in SAH patients, the AST:ALT (ratio) was also significantly predictive of CONUT [42], LTDH [43], and AUDIT [44], suggesting an overall role of chronic and recent heavy drinking along with poor nutrition in the progression of liver injury. This supports findings from earlier studies that poor nutritional status exacerbates liver damage in AH [45,46]. Dietary deficiencies, often present in chronic alcohol users, may impair liver function and repair mechanisms, leading to worsened liver injury [47].

The observation that low-risk SAH patients displayed the highest AST ratio, indicating more pronounced liver injury, and lower LTDH, suggests that even those with a lower immediate mortality risk can exhibit severe liver damage (Table 1). This finding challenges the conventional understanding that the severity of liver injury directly correlates with a high mortality risk. It indicates that early-stage severe AH can progress rapidly despite a seemingly lower mortality risk, emphasizing the need for early and aggressive intervention strategies.

AST and ALT levels were elevated in AH patients, with AST showing a higher ratio in severe AH (Group 2). This AST ratio is a well-established marker for liver injury severity [48]. The significant differences in bilirubin, INR, and albumin levels between Group 1 and Group 2 are consistent with their stratification by MELD scores, which incorporates these parameters [27]. Lower albumin levels in Group 2 reflect advanced liver dysfunction and impaired synthetic function [49]. These biomarkers may play a crucial role in precisely determining the stage of AH. One study found that the levels of M30 and M65 could distinguish between AH and AC, and that M65 levels, along with the apoptotic shift in M30/M65, were predictive of 30-day mortality in AH, with the M30/M65 ratio outperforming MELD and ABIC scores [50]. Another study discusses how K10M65 outperforms other severity/prognostic measures in AH [9], while another study favors K18 and deems biopsy unnecessary [51]. Th-17 cell-associated cytokines (IL-22 and IL-17) could also reveal the stage and course of AH treatment and pathology [52,53]. Adiponectin [54] and BMP6 [55] have also shown some level of biomarker response for ALD, but their exact role in AH needs to be ascertained. Gut microbial AH biomarkers have recently shown promise in the determination of AH diagnosis [56].

Our use of the Controlling Nutritional Status (CONUT) score revealed significantly higher scores in severe AH patients, highlighting the prevalence of malnutrition and its exacerbating role in liver injury [57]. An interesting observation was that severe AH patients in the low-mortality-risk group had higher CONUT scores than those with high mortality risk. A possible explanation for this could be a limitation of the ABIC risk stratification score, which prioritizes organ failure markers over nutritional status. Thus, a patient might score “low” on mortality risk due to preserved renal or coagulation function, despite being severely malnourished. Although malnutrition may not always be reflected in standard mortality scores, it still holds prognostic value as demonstrated by its link with increased morbidity and mortality across various liver diseases, including AH [31]. Elevated Maddrey and Child–Turcotte–Pugh (CTP) scores in Group 2 further confirm the utility of these scores in assessing disease severity and mortality risk [34].

Multivariable regression analyses identified LTDH, nutritional deficiency (CONUT score), and alcohol dependence (AUDIT score) as robust predictors of liver injury progression in severe AH patients. The AST to ALT ratio, strongly associated with these factors, has demonstrated its predictive value in assessing liver injury and mortality risk [48]. This finding underscores the importance of monitoring chronic alcohol consumption and nutritional status in clinical practice.

Our findings highlight the need for comprehensive clinical assessments in AH management, integrating alcohol use history, nutritional status, and disease severity markers. The early identification of high-risk patients, particularly those with severe AH and significant alcohol dependence, can guide timely interventions to mitigate liver injury and improve outcomes [58,59]. Future research should focus on validating these findings in larger cohorts and exploring therapeutic strategies targeting alcohol cessation and nutritional support to optimize patient management.

The outcomes support integrating chronic and recent drinking assessments and nutritional evaluations into clinical practice for AH patients [60]. The corresponding significant differences in MELD, Maddrey, CTP, and Lille scores across ABIC groups validate their importance in assessing disease severity along with prognosis estimation in AH patients (Table 1). The Maddrey score, a well-established predictor of short-term mortality, and the CTP score, which evaluates liver function and portal hypertension, were both significantly higher in the moderate- and high-risk ABIC groups, emphasizing their role in stratifying disease progression. The Lille score, which predicts early treatment response, differed significantly between the low- and moderate-risk as well as the low- and high-risk groups, highlighting its clinical utility in identifying early deterioration. Tailoring interventions based on these factors could enhance patient outcomes by addressing the multifaceted nature of liver injury in AH. For instance, targeted nutritional support and alcohol cessation strategies might mitigate the severity of liver injury and potentially improve survival rates.

Several scoring systems showed statistically significant differences across ABIC categories when reviewed based on the ABIC categories by themselves. The MELD score, Maddrey score, and Child–Turcotte–Pugh (CTP) score were significantly higher in the moderate- vs. high-risk group and the low- vs. high-risk group overall. The Lille score [61] showed significant differences between the low- vs. moderate-risk groups and the low- vs. high-risk groups overall. Age was significantly higher in the moderate- and high-risk ABIC groups, reflecting its direct role in ABIC calculation. This finding supports aging as a risk factor for worse liver outcomes, likely due to prolonged alcohol exposure and diminished hepatic regenerative capacity, further emphasizing its prognostic value in alcohol-associated hepatitis (AH). Males comprised a significantly higher proportion of the moderate- and high-risk ABIC groups, aligning with the well-documented higher prevalence of AH in men.

The alignment of the mortality and severity scoring systems with ABIC stratification in AH [62] supports their combined use in risk assessment and clinical decision-making. We observe such modifying effects in liver disease that are modified by other contributing factors, such as infection [63]. Chronic drinking was considerably higher in the moderate- and high-risk ABIC groups compared with the low-risk group. This suggests that prolonged alcohol exposure plays a central role in advancing liver dysfunction and increasing mortality risk [64]. The significant difference between the low- and high-risk groups further highlights the cumulative impact of long-term alcohol consumption on disease severity. The association between long-term/chronic drinking and ABIC validates the need to incorporate long-term drinking patterns into risk stratification models, as chronic alcohol exposure remains a fundamental driver of disease progression in AH.

This study provides insights for hepatologists and clinicians working with AH and comorbid AUD on why and how to collect detailed information [65]. While AH is a challenging disease to manage, it becomes concerning when the risk level of poor prognosis grows, especially in severe AH patients [66]. Evaluating baseline chronic and severe drinking assessments is not necessary, but it has been shown in our study that their collection can provide information on the risk assessment for these patients. While CMP, CBC, and coagulation are performed together daily according to clinical guidelines, assessing the CONUT score may not require any further laboratory involvement. A simple algorithm output of CONUT could inform clinicians about the nutritional status of patients. This will be helpful in the clinical management of the disease and in developing precise treatment courses.

This study has several limitations. We address the important ones and discuss their impacts as follows. Our study is limited by its observational design and relatively moderate sample size. Additionally, the cross-sectional nature of the one-time data collection does not allow for the assessment of long-term outcomes, including mortality rates or transplant evaluations/outcomes. All data were collected from a single site, albeit from several hospitals (VA and ULH hospitals), which may limit the geographic generalizability. Nonetheless, as this study was conducted in a tertiary care setting, the patients were admitted with referrals from an expansive region. Thus, the findings remain clinically relevant and applicable to similar healthcare environments. This study did not investigate any novel biomarkers; thus, investigating the translational endpoints was not within the scope of this study. The evaluation of this study aimed at sex dimorphism was a limitation due to the sample size. There was an imbalance in the distribution of sex between groups: Gr.1 included 8 females and 18 males, whereas Gr.2 included 13 females and 22 males. The lack of racial diversity in the cohort further limits the generalizability of the results to more heterogeneous populations. AST:ALT was used as a progression factor [58]. The progression of liver injury according to the AST:ALT should be considered in the context of other corresponding blood work, clinical presentation, and, importantly, liver imaging screens [67]; this is what we had assessed as well (and confirmed with live imaging at the time of this assessment). However, our study intended to use nutrition and drinking context to determine the pathological course; thus, AST:ALT was selected since it has been used historically to assess progression. Additionally, the low-risk group consisted predominantly of younger individuals compared with the high-risk group, potentially introducing age-related confounding. An unexpected limitation was the lower frequency of ascites in severe cases, which appears paradoxical. This may be due to the prior initiation of diuretics or recent paracentesis before enrollment in this study, potentially reducing the identification of ascites at the time of data collection. Future prospective studies with larger cohorts are necessary to validate these findings and explore the causal relationships between drinking history, nutritional status, and liver injury progression in AH, with the scope of investigating the underlying biomarker response from a translational perspective. Patients with low ABIC scores showed higher levels of liver injury markers; a prospective study to estimate liver cell death could reveal ongoing liver health status, given that ABIC does not directly reflect necroinflammation.

## 5. Conclusions

In conclusion, this study enhances our understanding of the interplay between drinking patterns and nutritional status, and the clinical presentation that could describe the prognostic outcomes in severe AH. These insights contribute to developing more effective management strategies tailored to individual patient needs, aiming to improve the survival and quality of life of AH patients.

## Figures and Tables

**Table 1:** Baseline assessment of demographics, drinking patterns, laboratory parameters, clinical parameters, nutritional parameters, liver disease severity markers, and survival/prognostic markers.

Measures	Group 1 (Non-Severe AH, MELD < 20)			Group 2 (Severe AH, MELD ≥ 20)				Between-Group *p*-Value
	Low Risk (ABIC < 6.71) (*n* = 10; 16.40%)	Moderate Risk (ABIC 6.71 to <9) (*n* = 16; 26.23%)	Total (*n* = 26; 42.62%)	Low Risk (ABIC < 6.71) (*n* = 6; 9.84%)	Moderate Risk (ABIC 6.71 to <9) (*n* = 20; 32.79%)	High Risk (ABIC ≥ 9) (*n* = 9; 14.75%)	Total (*n* = 35; 57.38%)	
Age (yrs.) ^a,b,c,d,e,f^	45.10 ± 5.70	56.50 ± 5.80	52.12 ± 7.99	32.17 ± 4.62	46.35 ± 9.34	54.78 ± 8.87	46.09 ± 11.17	0.023
BMI (kg/m^2^)	39.48 ± 11.27	31.07 ± 6.03	33.18 ± 7.70	28.78 ± 12.98	31.76 ± 9.02	33.54 ± 10.11	31.20 ± 9.93	0.625
Sex (F/M)	5/5	3/13	8/18	3/3	9/11	1/8	13/22	NA
Race (AA/Cau)	0/10	1/15	1/25	1/5	4/16	2/7	7/28	NA
	Drinking Patterns							
AUDIT score	30.00 ± 6.08	19.50 ± 9.61	23.00 ± 9.72	26.60 ± 3.36	23.91 ± 9.15	21.33 ± 6.03	24.21 ± 7.49	0.719
LTDH ^c^	15.67 ± 6.03	26.40 ± 11.06	22.38 ± 10.54	13.50 ± 4.80	21.91 ± 10.61	35.33 ± 14.22	21.81 ± 11.82	0.909
	Laboratory Parameters							
ALT (IU/L)	66.00 ± 51.13	60.31 ± 48.63	62.50 ± 48.67	45.17 ± 15.01	51.60 ± 59.33	59.89 ± 41.26	52.63 ± 49.24	0.440
AST (IU/L)	140.50 ± 81.07	123.81 ± 99.35	130.23 ± 91.42	168.50 ± 62.62	189.70 ± 301.14	146.11 ± 45.13	174.86 ± 226.23	0.351
Bilirubin (mg/dL) ^d,e,f^	4.22 ± 4.44	4.25 ± 4.12	4.24 ± 4.16	13.10 ± 7.73	13.65 ± 7.36	20.46 ± 7.16	15.30 ± 7.79	<0.001
Creatinine (mg/dL)	0.83 ± 0.47	0.73 ± 0.22	0.77 ± 0.34	0.60 ± 0.18	0.90 ± 0.37	1.79 ± 1.65	1.08 ± 0.96	0.122
INR ^d,e,f^	1.23 ± 0.27	1.45 ± 0.45	1.37 ± 0.40	2.07 ± 0.41	2.03 ± 0.44	2.76 ± 1.49	2.22 ± 0.87	<0.001
Albumin (g/dL) ^e,f^	3.29 ± 0.81	3.11 ± 0.73	3.18 ± 0.75	2.18 ± 0.57	2.43 ± 0.66	2.24 ± 0.56	2.34 ± 0.62	<0.001
	Survival/Prognostic Markers							
ABIC score ^a,b,c^	6.08 ± 0.49	7.37 ± 0.50	6.87 ± 0.81	6.1 ± 0.28	7.62 ± 0.63	9.86 ± 0.63	7.93 ± 1.40	0.001
	Nutritional Parameters							
CONUT ^c,e,f^	5.14 ± 2.27	5.83 ± 2.41	5.58 ± 2.32	10.00 ± 2.00	7.62 ± 2.19	7.57 ± 1.27	7.88 ± 2.05	0.001
	Liver Disease Severity Markers							
Meld score ^c,d,e,f^	13.50 ± 3.98	13.88 ± 4.43	13.73 ± 4.18	23.50 ± 1.87	24.60 ± 3.58	31.56 ± 6.84	26.20 ± 5.39	NA (<0.001)
Maddrey score ^d,e,f^	16.16 ± 13.83	28.17 ± 24.59	24.16 ± 21.94	60.48 ± 17.37	56.71 ± 23.09	99.65 ± 68.98	68.40 ± 42.57	<0.001
CTP score ^e,f^	7.80 ± 2.44	8.06 ± 1.88	7.96 ± 2.07	10.50 ± 1.38	10.80 ± 1.24	11.44 ± 1.01	10.91 ± 1.22	<0.001
Lille score ^c^	0.07 ± 0.03	0.23 ± 0.12	0.17 ± 0.12	0.08 ± 0.04	0.42 ± 0.29	0.58 ± 0.19	0.40 ± 0.29	0.043
AST/ALT ratio ^f^	2.84 ± 2.78	2.45 ± 1.70	2.60 ± 2.14	4.17 ± 2.35	3.52 ± 1.28	2.84 ± 0.90	3.46 ± 1.46	Trend (0.067)
	Clinical Parameters							
Ascites (Yes/No)	4/6	8/8	12/14	5/1	17/3	8/1	30/5	NA

BMI—Body Mass Index, LTDH—lifetime drinking history (in years), ALT—serum alanine aminotransferase, AST—serum aspartate aminotransferase, AST:ALT—ratio of the liver injury markers AST divided by ALT, CONUT—Controlling Nutritional Status (unit: numerical). ^a^ Statistically significant difference between the ABIC low- and intermediate-risk subgroups in Gr.1. ^b^ Statistically significant difference between the ABIC low- and intermediate-risk subgroups in Gr.2. ^c^ Statistically significant difference between the ABIC low- and high-risk subgroups of Gr.2. ^d^ Statistically significant difference between the ABIC intermediate- and high-risk subgroups of Gr.2. ^e^ Statistically significant difference between the ABIC low-risk subgroups of Gr. 1 and Gr.2. ^f^ Statistically significant difference between the ABIC intermediate risk subgroups of Gr. 1 and Gr.2.

## Data Availability

The datasets analyzed during the current study are available from the corresponding author upon reasonable request. The contact email ID of the author for data inquiry is v0vats01@louisville.edu.

## Abbreviations

The following abbreviations are used in this manuscript:

ABIC	Algorithm of “Age-Bilirubin-INR-Creatinine”
AH	Alcohol-associated Hepatitis
ALD	Alcohol-associated Liver Disease
AST:ALT	Aspartate transaminase: Alanine transaminase ratio
AUD	Alcohol use disorder
AUDIT	Alcohol Use Disorders Identification Test
BAC	Blood alcohol concentration
BMI	Body mass index
CONUT	Controlling Nutritional Status Test
CS	Clinically significant
CTP	Child–Turcotte–Pugh Score
CMP	Complete Metabolic Panel
CBC	Complete Blood Count
Lille	Lille Model for Alcohol-associated Hepatitis (Mortality Assessment at day 7)
LTDH	Lifetime drinking history, one-year drinking
Maddrey-DF	Maddrey’s Discriminant Function
MELD	Model for End-Stage Liver Disease
NCS	Clinically non-significant
